# Optimizing The Cell Seeding Protocol to Human Decellularized Ovarian Scaffold: Application of Dynamic System for Bio-Engineering

**DOI:** 10.22074/cellj.2020.6604

**Published:** 2019-10-14

**Authors:** Leila Mirzaeian, Farideh Eivazkhani, Maryam Hezavehei, Ashraf Moini, Fereshteh Esfandiari, Mojtaba Rezazadeh Valojerdi, Rouhollah Fathi

**Affiliations:** 1. Department of Developmental Biology, University of Science and Culture, Tehran, Iran; 2. Department of Embryology, Reproductive Biomedicine Research Center, Royan Institute for Reproductive Biomedicine, ACECR, Tehran, Iran; 3. Department of Endocrinology and Female Infertility, Reproductive Biomedicine Research Center, Royan Institute for Reproductive Biomedicine, ACECR, Tehran, Iran; 4. Department of Gynecology and Obstetrics, Arash Women’s Hospital, Tehran University of Medical Sciences, Tehran, Iran; 5. Vali-e-Asr Reproductive Health Research Center, Tehran University of Medical Sciences, Tehran, Iran; 6. Department of Stem Cells and Developmental Biology, Cell Science Research Center, Royan Institute for Stem Cell Biology and Technology, ACECR, Tehran, Iran; 7. Department of Anatomy, Faculty of Medical Science, Tarbiat Modares University, Tehran, Iran

**Keywords:** Mesenchymal Stem Cells, Ovary, Peritoneum, Seeding, Tissue Engineering

## Abstract

**Objective:**

Decellularized tissue scaffolds provide an extracellular matrix to control stem cells differentiation toward specific lineages. The application of mesenchymal stem cells for artificial ovary production may enhance *ex vivo* functions of the ovary. On the other hand, the scaffold needs interaction and integration with cells. Thus, the development of ovarian engineered constructs (OVECs) requires the use of efficient methods for seeding of the cells into the ovarian and other types of scaffolds. The main goal of the present study was to develop an optimized culture system for efficient seeding of peritoneum mesenchymal stem cells (PMSCs) into human decellularized ovarian scaffold.

**Materials and Methods:**

In this experimental study, three methods were used for cellular seeding including rotational (spinner flask) and static (conventional and injection) seeding cultures. OVECs were evaluated with Hematoxylin and Eosin staining and viability analyses for the seeded PMSCs. Then, immunohistochemistry analysis was performed using the best method of cellular seeding for primordial germ cell-like cells, mesenchymal stem cells and proliferation markers. Stereology analysis was also performed for the number of penetrated cells into the OVECs.

**Results:**

Our results showed that rotational seeding increases the permeability of PMSCs into the scaffold and survival rate of the seeded PMSCs, comparing to the other methods. On the other hand, rotationally seeded PMSCs had a more favorable capability of proliferation with Ki67 expression and differentiation to ovarian specific cells with expression of primordial germ cell line markers without mesenchymal stem cells markers production. Furthermore, stereology showed a more favorable distribution of PMSCs along the outer surfaces of the OVEC with further distribution at the central part of the scaffold. The average total cell values were determined 2142187 cells/mm^3^ on each OVEC.

**Conclusion:**

The rotational seeding method is a more favorable approach to cell seeding into ovarian decellularized tissue than static seeding.

## Introduction

Tissue engineering techniques provide a suitable decellularized extra cellular matrix (ECM) for renewal of functions of impaired tissues. It has been assumed that low levels of unfavorable immune response due to lack of cells leads to use decellularized ECMs as a practical technique especial in the case of allo and xeno-transplantation (1). ECM, consists of complex enclosed compositional and architectural elements, depending on the tissue source, and directly determines the cellular fate map (2, 3). ECM ultrastructure facilitates penetration of selected cell types (4), and modulates the migration of cells into the scaffold and influences tissue specification, cell morphology and differentiation potential (5, 6). Mesenchymal stem cells (MSCs) are favorable candidates to be used in tissue engineering and regenerative medicine for secretion of paracrine factors, ovarian damage treatment and no immunogenicity (7-11). Among the sources of mesenchymal cells, the peritoneal mesothelium has specific properties such as plastic adherence, self-renewal, appearance of MSCs surface markers and differentiation potential to mesoderm and nonmesoderm cell lines called peritoneum mesenchymal stem cells (PMSCs) (12-14).

Regenerative medicine is increasingly gaining importance in the treatment of female infertility. The production of ovarian engineered constructs (OVECs) can potentially restore fertility in women with ovarian dysfunctions like premature ovarian failure (POF) and ovarian cancer or postmenopausal re-fertility. Scientists believe that decellularized tissue scaffolds have a microenvironment for MSCs, which allows them to differentiate into tissue specified cells. Thus, it seems promising to introduce cells into the decellularized ovarian ECM in women for restoration of female fertility. This is challenging and requires development of optimized methods for seeding MSCs into ovarian scaffolds and making an organoid.

The use of static culture systems such as tissue culture petri dishes for MSCs seeding is simple, rapid and well established, but has serious limitations such as variability and user dependence. Furthermore, seeding efficiency under this environment is as low as 10-25%. Thus, it is important to extend and develop alternative systems like stirred vessels or rotational seeding to minimize cell death and increase cell penetration. This particular seeding technique has been reported to increase the seeding efficiency to approximately over 90% (15). The rotational seeding method holds promise for the development of artificial ovaries.

In the current study, human decellularized ovarian scaffold was used as a natural bed for cell attachment, penetration, expansion and differentiation to tissue specific cells. Rotational and static seeding methods were used to compare migration and distribution of the PMSCs within the human decellularized ovarian scaffold, to evaluate their survival rate and differentiation potential into ovarian cell-like cells.

## Materials and Methods

### Human ovarian tissue decellularization

In this experimental study, all the steps were designed to abide by the rules of research Ethics Committee of Royan Institute (IR.ACECR.ROYAN.REC.1396.67). Ovarian tissue was collected from trans-sexual humans. Ovarian tissue was trimmed into 2 mm-thick sections of cortex and medulla. In order to decellularize human ovarian tissue slices, the samples were stored at -80˚C overnight and then placed and agitated in 0.5 M NaOH solution at room temperature, overnight. Tissue slices were finally treated with a nuclease supplemented solution (RNase/DNase, Thermo fisher, USA) and washed in sterile phosphate buffered saline (PBS, Invitrogen, USA) for 48 hours with 6 times exchange.

### Scanning electron microscopy study

For scanning electron microscopy (SEM) analyses, some of the samples were fixed with a fresh prepared 2.5% glutaraldehyde solution (Sigma, USA) at 4˚C for 24 hours. The samples were immersed into PBS overnight and fixed in 1% osmium tetroxide (Sigma, USA) at 25˚C for 2 hours. Dehydration was performed with ethanol at ascending concentrations of 30, 70, 80, 90, and 100%. After mounting on aluminum foil, the samples were covered with a gold layer. Then the structures of samples were investigated using a SEM (VEGA\TESCAN, Czech Republic).

### Seeding of peritoneum mesenchymal stem cells into human decellularized ovarian scaffold

**Fig 1:**
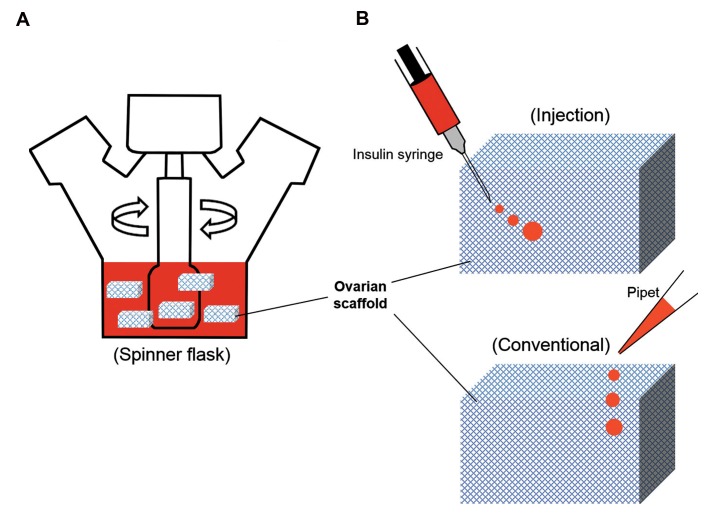
Cell seeding protocols. **A.** Rotational seeding: scaffolds are located in a spinner flask with 100ml volume of cell suspension and **B.** Static seeding: cell suspension is transferred directly into the human ovarian scaffold with insulin syringe (G-27) or onto the outer surfaces of the scaffold with pipet.

In our previous study, we isolated and characterized PMSCs and demonstrated their differentiation abilities into ovarian cell-like cells (14). Briefly, Peritoneum mesothelial was separated from mouse anterior abdominal walls and washed with PBS. Tissue fragments were split into smaller pieces and cultured in Dulbecco’s modified Eagle’s medium/ F12 (DMEM/F12, Gibco, USA) supplemented with 15% fetal bovine serum (FBS, Gibco, USA), 1% non-essential amino acids 100× (Gibco, USA), 1% insulin transferrin selenium (ITS, Gibco, USA), 1% Glutamax (Gibco, USA), 1% (5000 U/ml) penicillin/ streptomycin (Thermo fisher, USA) under 21% O_2_, 5% CO_2_, 97% humidified atmosphere at 37˚C. PMSCs were expanded on the basis of plastic adherence in cell culture T25 flasks for 8 passages. Two separate approaches were applied for PMSCs seeding into human decellularized ovarian scaffold. To compare the influence of rotational or static seeding on the tissue-engineered ovarian construct, the decellularized ovarian scaffolds were divided into three groups as follows: 1) Rotational culture by spinner flask, 2) Static culture by conventional protocol, and 3). Static culture post cell injection (Fig .1). For this purpose, human ovarian decellularized ECM pieces (5×5×1 mm3) stored at -20˚C, were washed three times in PBS, then sterilized using 70% ethanol (2 hours) and ultraviolet (20 minutes) and soaked in the culture medium for 1 hour at 37˚C under a 5% CO_2_, humidified atmosphere. In group 1, PMSCs expansion was performed using of 100 ml spinner flask with stirring speed of 20 rpm to support cellular adhesion and distribution into exterior and interior surfaces of the scaffolds. In each repetition, six scaffolds were placed in each spinner flask plunged in 2×10^6^ cells per scaffold (2×10^6^ cells/ scaffold). The volume of culture medium was 100 ml (Fig .1A). In group 2, the same number of PMSCs suspended in a solution, were injected with an insulin syringe (G-27) into different parts of the scaffold and in group 3, PMSCs were placed on sample surfaces with the same culture medium, in 12 well tissue culture plates covered with 1% agarose gel (Fig .1B). All samples were cultured at 37˚C in 5% CO_2_ for 1 week and 50% of the medium was replaced every 3 days. In each group, three tissue constructs were fabricated and used for histological characterizations and cell viability analyses. Finally, after finding the optimum seeding protocol, immunohistochemistry staining and stereology tests were carried out to the most appropriate group to assess proliferation and penetration ability of the seeded PMSCs .

### Cellular viability

OVECs were observed under light microscope (Olympus CKX41, Japan). Analyses of the viable or metabolic activated cells seeded into exterior and interior surfaces of the OVECs were made using 3-(4,5-dimethylthiazol-2- yl)-5-(3-carboxymethoxyphenyl)-2-(4-ulfophenyl)-2Htetrazolium (MTS) assay. For this purpose, the OVECs were transferred to a new well after being washed in PBS for 3-4 hours. Then, the constructs were incubated with 100 μl of the DMEM/F12 free medium supplemented with 20 μl of MTS/PMS, which produced a color response in the presence of viable cells. To avoid the effect of the matrix on spectrophotometry, 100 μl of the reaction medium from each well was relocated to another well. Cell viability was recorded as absorbance at 490 nm by microplate reader (thermo scientific, America).

### Histology assessments

OVECs derived from three seeding protocols were fixed for Hematoxylin and Eosin (H&E) staining, in Bouin’s (24 hours), and then they were transferred to 10% buffered formalin (24-72 hours). The OVECs were then washed, processed and embedded in paraffin wax. The blocks were serial sectioned to 6 μm thickness using a microprocessor machine (Thermofisher, USA). The sections were labeled on glasses and stained with H&E. All images of OVECs cross sections were captured on an upright microscope (Olympus IX51, Japan) and cellular distribution and migration of seeded PMSCs were evaluated.

### Immunohistochemistry for Stella, Prdm14, Blimp1, CD90 and Ki67

In order to assess PMSCs differentiation into primordial germ cell-like cells in cultured OVECs in spinner flask, developmental proteins were detected by immunohistochemistry. For this purpose, sections (with 6 μm thickness) were placed on charged slides, in 60˚C for 30-40 minutes. After deparaffinization and hydration, sodium citrate buffer (PH=6) was applied for 30 minutes at 90˚C to retrieve masked antigens. Then sections were immersed into PBS-tween (0.05%). For peroxidaselinked immunostaining, endogenous peroxidase was removed by 10% hydrogen peroxide (H_2_O_2_) for 30 minutes and rinsed twice in PBS-tween. Then, Triton X-100 (0.5%) was used for 15 minutes for membrane permeability. Non-specificities were blocked with 10% secondary host serum at 37˚C for 1 hour and rinsed twice in PBS-tween. Primary antibodies were diluted in 10% secondary host serum and PBS (one to one ratio) and incubated overnight at 4˚C. Purchased primary antibodies were anti-Stella (1:100, Santa Cruz, USA), anti-Prdm14 (1:100, Abcam, USA), anti-Blimp1 (1:100, Cell signaling, USA) anti-CD90 (1:100, BD, USA) and anti-Ki67 (1:100, Biolegend, USA). Subsequently, the sections were washed thrice with PBS-Tween, and incubated for 1 hour with one secondary antibody. Peroxidase-conjugated goat anti-rabbit (1:500, Invitrogen, USA) and rabbit antigoat (1:500, Abcam, USA) IgG antibodies were used and washed thrice with PBS-Tween and treated with the diaminobenzidine (DAB) reagent (ABC, detection IHC kit) in the dark at room temperature for 5-20 minutes. In the presence of peroxidase enzyme, DAB produces a brown precipitate. Negative control was made by incubating the sections only with secondary antibodies. Washing was repeated and sections were counterstained with hematoxylin. Sections were mounted under coverslips, dried overnight, dehydrated, cleared and observed under light microscopy (Olympus IX51, Japan). The percentage of positive cells was calculated by counting the number of brown-stained cells versus the number of hematoxylinpositive nucleus, representing the total cell numbers.

### Estimating cell number and migration by stereology

**Fig 2:**
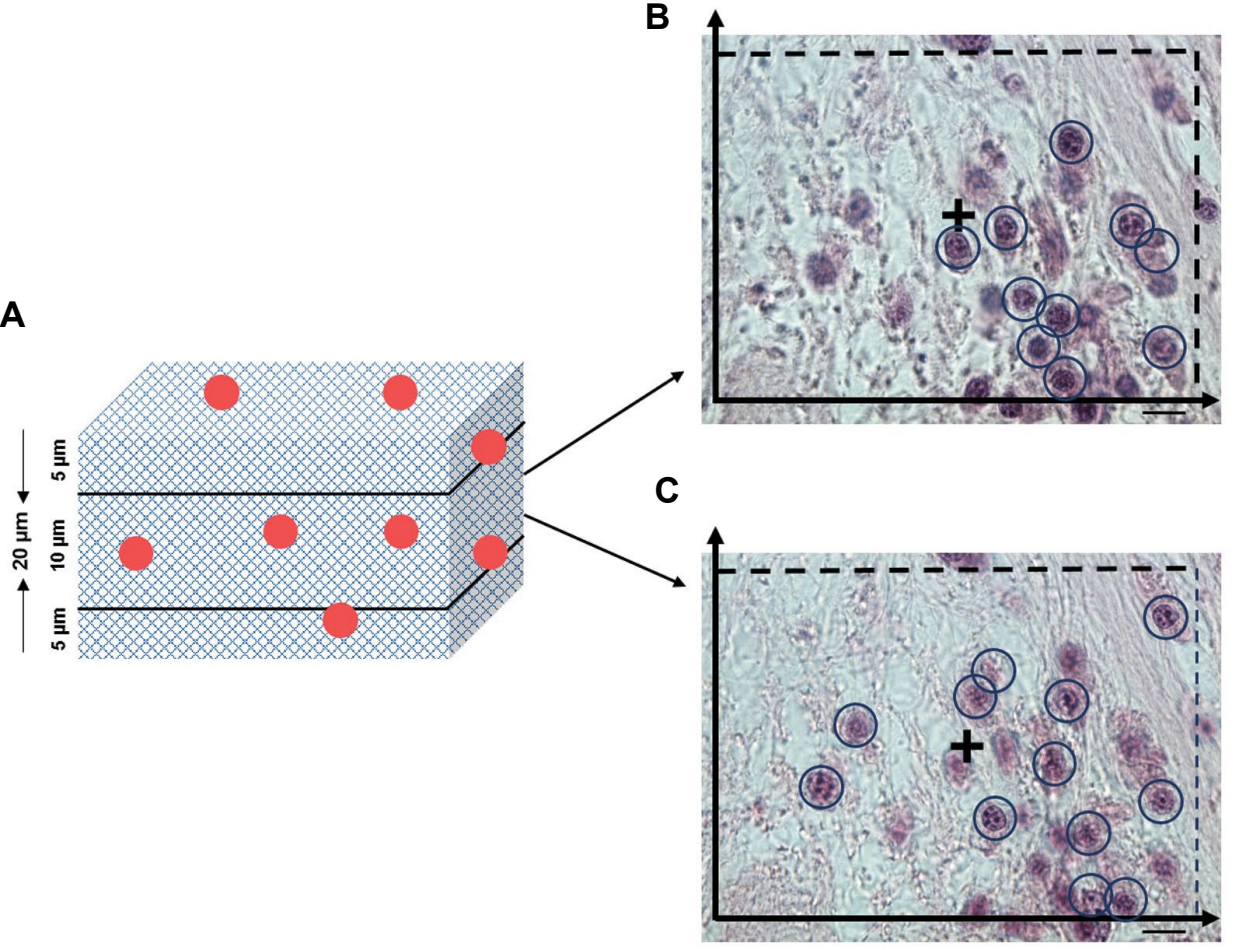
Cell number estimation in OVECs by using the optical dissector. **A.** Two regions of the gourd zone with 5 μm from the up and down areas of the sections are not counted, **B,** and **C.** Two different depths of 10 μm in which the count is performed. An unbiased counting frame of area superimposed on each sampling field used to sample the cell nucleoli (scale bars: 10 μm).

Stereological methods produced 3D results from 2D images of OVECs. To calculate the number of PMSCs, an optical dissector was used. In this method, fixed OVECs cultured in rotational seeding were embedded in paraffin block. The technique was used to achieve isotropic uniform random (IUR) sections. The paraffinized scaffolds were serially sectioned to 20 μm thickness (H&E staining) for cell number estimation. The selected sections were studied using an upright microscope (×100 magnification) and a microcator (ND 221 B, Heidenhain, Germany) connected to a computer for measuring the Z-axis travel. The nuclei of PMSCs were observed using an unbiased counting frame covered on the monitor. Any nucleolus derived from maximal focus was selected if it was placed in the counting edge or touched the inclusion edge and did not touch the exclusion boundaries (Fig .2). Finally, the following formula was used to calculate the numerical density of the cells: Nv (cells)=[ΣQ/(a/f×ΣP×h)]×V, in which “ΣQ” is the total number of the counted cells, “Σp” is the total number of the points superimposed on the selected fields, “h” represents the tissue thickness and “a/f” stands for the frame area in the tissue actual scale. To obtain the total cell number, the results were multiplied by the total volume (V) of the scaffold (16).

### Statistical analysis

The results were reported as the mean ± SEM and was conducted by using three technical and biological replicates. Statistical analyses were performed using SPSS software (version 21, IBM, USA). Analysis of variance was carried out and data were subjected to oneway ANOVA test, followed by the Tukey test. P<0.05 was considered as significant.

## Results

### Morphology characterization of ovarian engineered constructs

**Fig 3:**
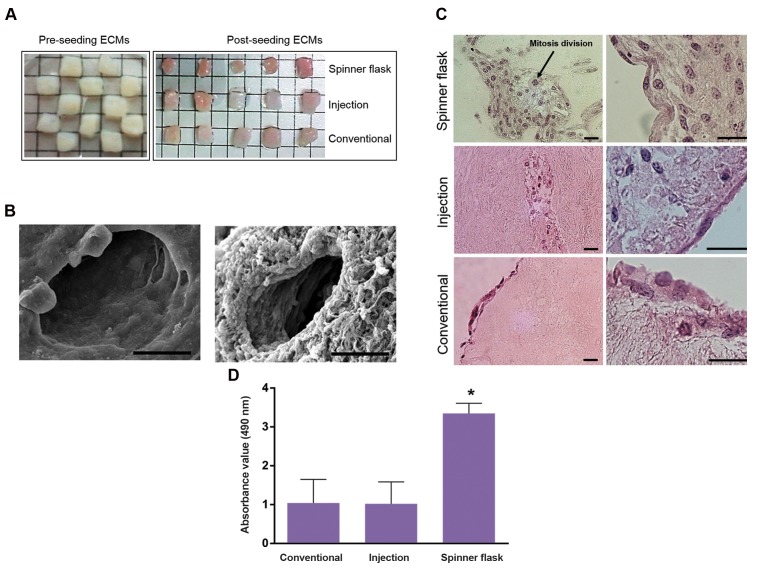
Comparison of seeding protocols. A. Morphological, B. SEM, C. H&E staining, and D. MTS analyses. Comparison of recellularized human ovarian ECM with PMSCs through 3 seeding protocols (scale bars: 25 μm). ECM; Extra cellular matrix, MTS; 3-(4,5-dimethylthiazol-2-yl)-5-(3-carboxymethoxyphenyl)-2- (4-ulfophenyl)-2H-tetrazolium, SEM; Scanning electron microscopy and *; P<0.05.

OVECs produced by rotational seeding were red in color, but in static seeding, they were paled (Fig .3A). Also, SEM shows that the pore size without and with ovarian cells was approximately 50 μm and this allowed the cells to penetrate into the scaffold (Fig .3B). In order to evaluate and compare the attachment and infiltration of PMSCs into human decellularized ovarian ECM, H&E staining was carried out for serial sections obtained from OVECs. In rotational seeding, PMSCs penetrated not only into the exterior surfaces also to the depth of the scaffolds and mitosis divisions were seen as well. Active division increased the cell number on tissue periphery and PMSCs expanded in the scaffold. On the other hand, the cells and nuclei exhibited appropriate morphology and alignment (Fig .3C). But in static (injection) seeding, few cells were evident via H&E staining in marginal parts of the tissue and morphology of PMSCs seeded into decellularized ECM did not represent well condition. In conventional seeding, PMSCs not only did not penetrate to decellularized scaffold, also they could not migrate into the ECM clefts. In addition, in static seeding, the presence of many grooves in decellularized scaffold may indicate the separation of the components. Generally, rotational seeding increased cell seeding penetration and uniformity of the decellularized scaffold more than the other methods (Fig .3C).

### Viability of seeded peritoneum mesenchymal stem cells

Mean viability rate of PMSCs seeded into the scaffolds measured by mitochondrial activity, in rotational seeding were significantly (P<0.05) more than both static seeding methods. In addition, the expression of ki67 as a proliferative marker confirmed that rotational seeding method retains proliferation ability of PMSCs in addition to increasing their survival rate (Fig .3D). Cell division after applying this technique verified the previous observation (Fig .3C). Therefore, it could be concluded that the rotational seeding technique resulted in more suited recellularized construct.

### Immunohistochemistry and cell differentiation

**Fig 4:**
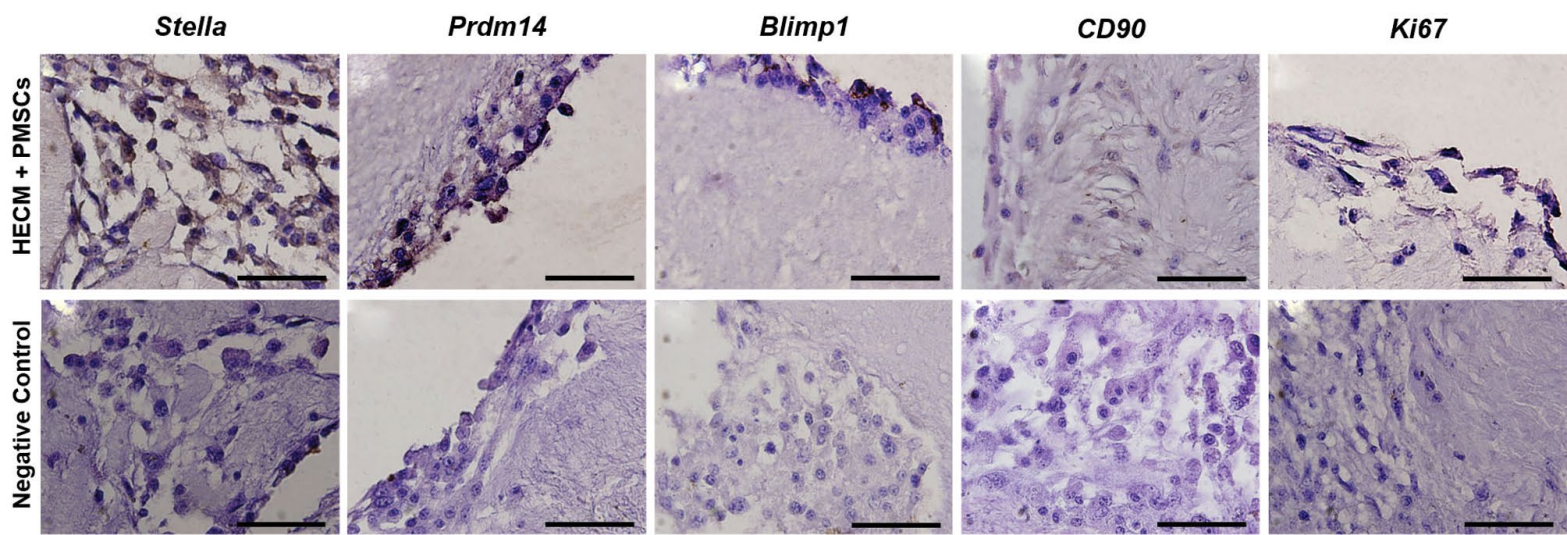
Immunohistochemical analysis of recellularized human ovarian matrix with PMSCs through spinner flask for antibodies against germ cell markers (Stella, Prdm14 and Blimp1) mesenchymal stem cell (CD90) and proliferation markers (Ki67) (scale bars: 20 μm).

The results of H&E staining and viability analysis showed that the spinner flask generated the best results of cell seeding more than static methods. Thus, the OVECs derived from rotational technique were processed for cell phenotype characterization using immunohistochemistry staining. Figure 4 shows that the seeded cultured cells displayed a primordial germ cell-like cells properties by expressing Stella, Prdm14, and Blimp1 (Fig .4A-C). The low expression of mesenchymal stem cell marker (CD90) in PMSCs seeded into OVECs indicates a decrease in mesenchymal features in these cells (Fig .4D). On the other hand, production of proliferation protein (Ki67) shows self-renewal in seeded cultured PMSCs (Fig .4E). There was no positive staining in the negative controls (Fig .4F, J), showing that the secondary antibodies labeled to specific antigens.

### 3D cell distribution in scaffolds cultured under rotational condition

**Fig 5:**
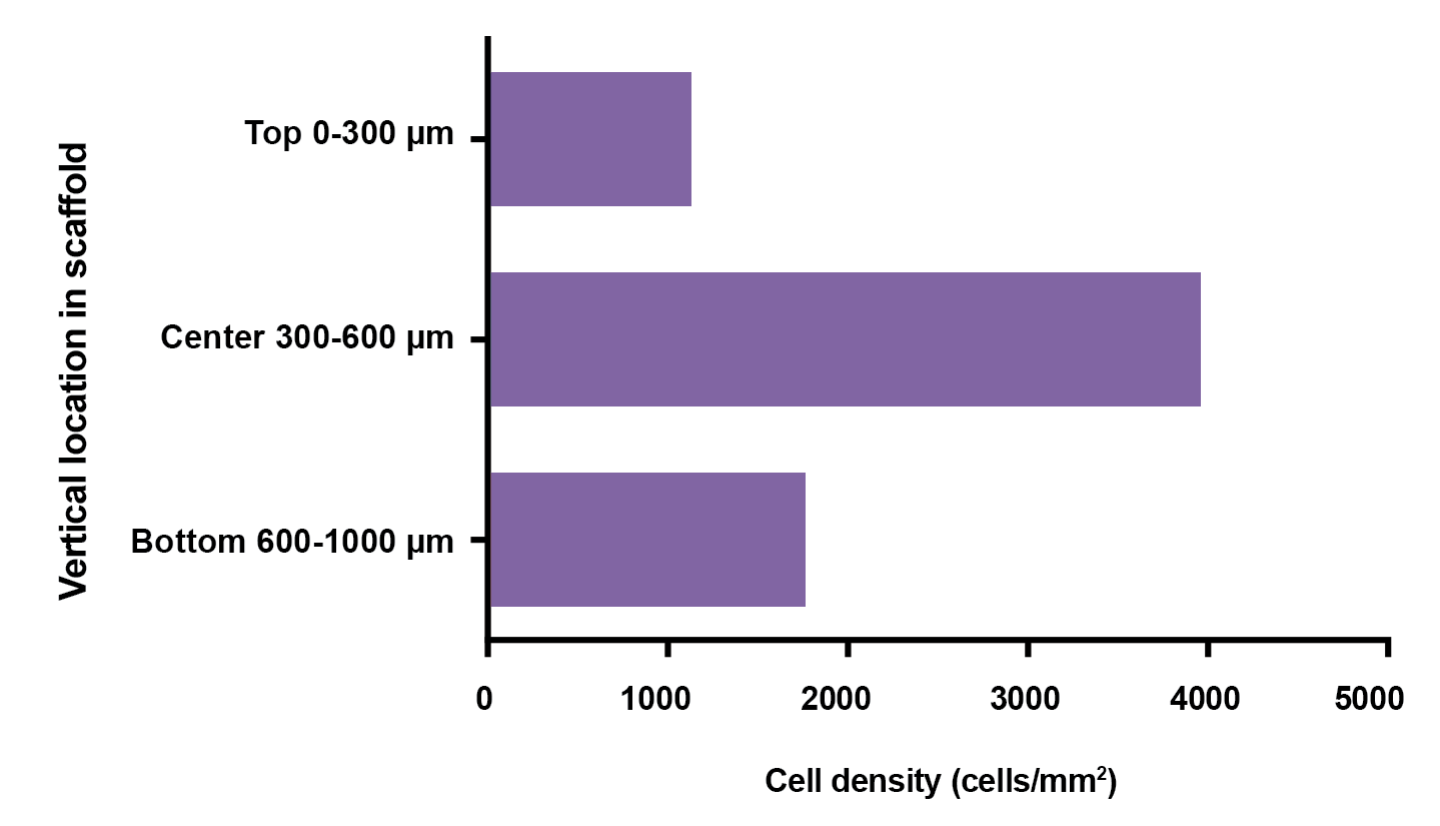
Cross-sectioned counted cell density in OVEC (n=1). OVEC was divided into three equal zones representing the exterior outer surfaces, the center of the section and the bottom zone. From the seeded surface, the cell density increased with increasing depth to the center and decreased below to the lower exterior surface.

The cellular distribution and density were evaluated within the OVEC produced by rotational seeding method after 7 days of incubation. This method led to PMSCs distribution throughout the ovarian decellularized ECM. The seeding efficiency of rotational method (spinner flask) was 53.5%. Total cell values on the OVEC were determined to be 2142187 cells/mm^3^. The images showed more favorable distribution of PMSCs throughout the peripheral parts of the tissue sections. Vertical delivery of the cells in the crosssection showed low density of cells (1133 cells/mm^2^) on the upper part of the scaffold. The interior part of the scaffold showed improved cell penetration, with cell density of 3960 cells/mm^2^ for the central zones. The cell density diminished slightly at the lowest surface of the scaffold, at 1762 cells/ mm2 density (Fig .5).

## Discussion

 Ovarian tissue cryopreservation is one of the strategies to fertility preservation of cancer affected women. Tissue engineered ovaries from decellularized ovarian scaffolds can prevent reintroduction of malignant cells and lead to development of a transplantable scaffold. Decellularized ovarian scaffolds could be recellularized with MSCs and implanted after appropriate *ex vivo* regeneration steps. This technology also can apply to women with POF. In the present study, tissue engineering was used for primarily recellularization of human decellularized ovarian scaffold with mouse PMSCs. We obtained ovarian decellularized scaffolds from trans-sexual human ovaries that preserved their natural properties and showed retention of main ECM structure in SEM.

Many techniques are used for cellular seeding into whole organ or tissue segment scaffolds but the best protocol for PMSCs seeding into 5×5×1 mm segments of ovarian scaffold must be chosen. The effects of rotational and static seeding protocols on cell repopulation and arrangement beside of cell permeability level, morphology and viability were evaluated and compared after 1 week of *in vitro* culture. H&E staining showed penetration of fewer PMSCs in the static seeding method without cellular arrangement but the rotational seeding promoted cell repopulation deep into the ovarian scaffold. Therefore, static culture protocols (conventional and injection) have serious limitations for cellular seeding. On the other hand, the porous structure of the decellularized ovarian scaffold as shown by SEM causes cell leakage during injection and the lack of medium flow leads to the absence of cellular entrance into scaffold in the conventional method.

Our results showed that the rotational culture system using a spinner flask has many advantages. It supports cell alignment and stimulates OVECs formation. The first recellularization attempts of decellularized ovaries by Laronda et al. (17) were made using mouse conventional ovarian cells seeding into bovine decellularized ovary for 2 days. Low-speed rotational seeding plays an important role to increase the efficiency of early cell seeding, stimulate cell adhesion, differentiation and construct development. In the present study, spinner flask operating at a speed of 20 rpm was able to preserve cell viability, proliferation and differentiation. However, the efficiency of cellular proliferation and differentiation rates are still low. Rotational seeding homogenizes culture medium and may induce transient oxygen and supplements and this, in turn, can increase the quantity and distribution of cells in the decellularized ovary. Wang et al, indicated that rotational MSCs seeding was more effective than static tissue culture in oxygenation of the recellularized myocardial scaffolds (18). Moreover, immunohistochemistry staining for the OVECs confirmed that rotational seeding generated positive tissue remodeling.

It seems that the attachment of PMSCs to ovarian ECM leads to cell and tissue interaction signals. It is believed that peritoneum mesothelial cells have a common embryonic origin with ovarian surface epithelium (OSE) cells (19). Bukovsky et al. (20) displayed that OSE cells can be a bipotent source for granulosa and germ cells. Therefore, PMSCs, both in terms of location and origin are more likely to differentiate into ovarian celllike cells than other MSCs. As in our previous study, we have displayed the differentiation potential of PMSCs in human follicular fluid and cumulus cell conditioned media into ovarian cell-like cells *in vitro* (14). Our results in this study showed that the differentiated cells have primordial germ cell-like cells phenotypes through expression of Stella, Prdm14, and Blimp1 proteins. These markers cause proliferation and migration induction in primordial germ cells (21). Stella plays a significant role in maturing oocytes and preimplantation embryos (22). This protein may be involved in germ line determination in ovarian ECM. Furthermore, Ki67 as a proliferation protein was observed in one-week cultured OVECs.

Cortiella et al. (23) compared the influence of decellularized lung, gelfoam, Matrigel, and collagen I hydrogel matrices on mouse embryonic stem cells attachment, differentiation and formation in a tissue complex. A rotational approach was used for cellular seeding and the results showed that decellularized lung scaffold had improved cell preservation with more differentiation rate of embryonic stem cells into epithelial and endothelial cells than those of others. It is believed that rotational seeding culture decreases stress and maintains a steady flow of nutrients to the developing constructs. Collectively, rotational seeding data showed that ovarian scaffolds are likely to have the necessary signals to support initial attachment, proliferation and differentiation. Ji et al. (24) also showed that a dynamic culture system was more favorable than static culture in improving seeding of mouse bone marrow mesenchymal stem cells into rat liver scaffold by creating optimal stream rate and led to significantly advanced proliferation. Scaffold induced lineage-specific differentiation hepatocyte-like cells from MSCs. Moreover, Vermeulen et al. (25) showed that pig immature decellularized testicular scaffold is able to support human sertoli cells attachment, proliferation and functionality. Extremely low expression or even lack of expression of CD90 as a MSCs surface marker in our study confirmed that rotational seeded and attached PMSCs into ovarian ECM, lost mesenchymal properties. However, the nature of mesenchymal stem cells were confirmed in the cells by immunoflorosent assay before cell injection into the ECM.

Santos et al. (26) believed that microcarrier-based stirred culture system subjected to an agitation, affects the cell surface antigens and reduces CD90 expression in human adipose tissue stem cells. It seems that prolonged agitation time to 2 weeks in the mentioned study led to alteration in surface markers expression. Therefore, our suggestion is that mesenchymal properties of PMSCs may change belong to their attachment to ovarian ECM and their differentiation. Woloszyk et al. (27) showed that mineralized matrix formation potential in human dental pulp stem cells seeded and grown on porous 3D silk fibrin scaffolds, is enhanced in the rotational culture system. Furthermore, Xue et al. (28) showed that rat adipose tissue derived stem cells can attach, grow and differentiate to vascular endothelial and tubular cells in rat decellularized kidney scaffold.

There is a need to develop a simple, effective and inexpensive method for evaluation of cell distribution and penetration rates into scaffolds. We calculated the total number and density of distributed PMSCs into OVECs by the stereological method. There have, hitherto been few studies on the measurement of seeded cell density and distribution in tissue-engineered constructs. Thevenot et al. (29) investigated the permeability and distribution rate of fibroblasts in a variety of seeding protocols. They concluded that dynamic seeding technique facilitates moving a cell solution along the scaffold and leads to cell penetration into the scaffold pores, as well as on the outer surfaces. In comparison, the efficiency of rotational seeding in our study was 53.5%, which is almost equal to centrifuge seeding efficiency (52%) as reported by Thevenot et al. (29). Finally, in the current study, the use of stereology helped find actual and accurate cell seeding ability data and showed that rotational seeding technique leads to a wide distribution of PMSCs on ovarian exterior surface as well as deep penetration into the center of OVEC.

## Conclusion

The use of spinner flask causes PMSCs movement around the ovarian scaffolds and enhances contact between the cells and the scaffold. This makes it a more favorable technique for cell seeding into decellularized ovarian tissue than conventional and injection methods.
